# Changes in Quality Traits and Oxidation Stability of *Syzygium aromaticum* Extract-Added Cooked Ground Beef during Frozen Storage

**DOI:** 10.3390/antiox11030534

**Published:** 2022-03-11

**Authors:** Mohammad Ashrafuzzaman Zahid, Jeong-Uk Eom, Rashida Parvin, Jin-Kyu Seo, Han-Sul Yang

**Affiliations:** 1 Department of Nutrition and Food Technology, Jashore University of Science and Technology, Jashore 7408, Bangladesh; zahid_24jstu@yahoo.com (M.A.Z.); rakhiparvin@yahoo.com (R.P.); 2 Division of Applied Life Science (BK21Four), Gyeongsang National University, 501 Jinju-daero, Jinju 52828, Korea; diawjddnr7@naver.com (J.-U.E.); tjwlsrb7942@gmail.com (J.-K.S.); 3 Institute of Agriculture and Life Science, Gyeongsang National University, 501 Jinju-daero, Jinju 52828, Korea

**Keywords:** cooked ground beef, *Syzygium aromaticum* extract, frozen storage, quality traits, oxidative stability

## Abstract

This study was accomplished by comparing the oxidative stability of (0.1%) *Syzygium aromaticum* extract (SAE) and (0.02%) butylated hydroxytoluene (BHT)-added cooked ground beef with an antioxidant free-control sample during frozen storage. All samples showed a non-significant (*p* > 0.05) effect on pH, thawing loss, redness, and yellowness values during storage. Incorporation of BHT and SAE led to a significant (*p* < 0.05) reduction in thiobarbituric acid-reactive substances (TBARS) and volatile levels as an active antioxidant. The generation of less volatiles found in SAE-treated samples up to 6 months (*p* < 0.05) of storage. Therefore, SAE-protected ground beef can lead to lower lightness, lipid oxidation, and volatile compounds levels after cooking compared with control and BHT samples.

## 1. Introduction

Meat and meat products are significant sources for nutritional components in human daily diet and are the most preferred food products considering broad consumption as fast meals over the past years because of a radical change in lifestyle. During the process and preservation for meat products, the oxidative process induces the degradation of lipid, protein, and color pigment, which may result in degradation for color, flavor, texture, and nutritional value in the meat and meat products [1]. Ground meat is further vulnerable to oxidation than whole meat cuts because of the larger surface area that permits straight interaction between lipid and air, and the better accessibility of oxidation promoters such as the released heme and non-heme iron from meat pigments hemoglobin, myoglobin, and phospholipids from disrupted cells [2]. Nevertheless, frozen storage is the most effective storage method to preserve the qualities of meats and meat-based products for a longer period. The degradation of quality characteristics is due to the physicochemical procedure. As a consequence, several investigations have revealed that the oxidative process is the main factor to lose the quality characteristics of different meats and meat products throughout frozen storage [3,4].

Lipid oxidization is regarded as the irrevocable, unavoidable, and complicated procedure which happens in food-matrix over the process and storing period, and maintains significant concerns in regards to a loss for quality traits, nutrient content, and economical interests [5]. Moreover, lipid oxidation becomes implicated as the major cause to decrease the quality such as oxidative stability and shelf-life for meats and meat products [6], and it is the main cause of deterioration of cooked meat, which is more susceptible than raw meat to lipid peroxidation while chilled and frozen [7]. Therefore, it is required to decrease these oxidative changes by adding antioxidants to meat and meat products to maintain the quality during storage. The use of antioxidants is evidenced as being a great policy to slow, retard, or hinder the oxidative process, preserve quality traits from rancidness, and improve color stability [8,9].

Butylated hydroxytoluene (BHT), which is a known synthetic antioxidant having the ability to scavenge peroxyl radicals and control free radical generation, has been used for retarding or inhibiting oxidation and increasing the shelf-life for meat products [10]. It allowed utilizing BHT at 0.02% in meat and meat products, and its usage indicates the level of packed meat for oneself [11]. Nevertheless, due to the causes of anti-nutritional, toxicological, and carcinogenic effects of synthetic antioxidants, there is increasing consumer demand for natural antioxidants [8,12]. In this contradictory case for large extent usage in meat products and consideration of health, synthetic antioxidants have been proposed for replacement by natural antioxidants from plant sources such as fruits, vegetables, and spices in the meat industry [13,14].

The extract of *Syzygium aromaticum* L. (SAE) belonging to the family of Myrtaceae was obtained from the dried flower buds, and have been extensively utilized in the food-processing industry regarding its unique flavor and advantageous health qualities. The antioxidant properties of SAE have been exhibited owing to the existence of phenol-based bioactive elements e.g., tannins, triterpenoids, and sesquiterpenes [15]. *S. aromaticum* extract (SAE), isolated from the dried flower buds, contains two major phenolic bioactive components such as eugenol and eugenol acetate that show potential antioxidative activities by scavenging hydrogen peroxide and reducing ferric ions [16]. In this sense, the incorporation of SAE as natural antioxidants may effectually restrain the oxidizing process for lipids and proteins, stabilize color value, improve sensorial traits, and expand the storage life for meat and meat products [9,17].

Over recent years, the usage of natural antioxidants has been raised to increase the oxidation stabilization for meat products. As an illustration, SAE was recorded for being a potential antioxidant to prevent the oxidative process in meat products [17]. Nevertheless, BHT is usually employed for showing antioxidative actions by the meat-processing industry [12]. Taking this into account, SAE and BHT have been incorporated in ground beef, and the comparative analysis for such antioxidant’s effects on oxidative stability, volatile compounds, and color values in cooked ground beef has been assumed.

Therefore, the objective of this study was to compare the antioxidant effect of SAE as natural antioxidant with a synthetic antioxidant such as BHT for cooked ground beef, by measurement of pH, thawing loss, thiobarbituric acid-reactive substances (TBARS), volatile compounds, and color values during 6-month frozen storage.

## 2. Materials and Methods

### 2.1. Syzygium aromaticum Extracts and Sample Preparation

The dried flower buds or *S. aromaticum* employed for the analysis were purchased from the provincial market. The dried bud was ground into fine powder and the reflux extraction method [18] was used for the separation of extract from powder. The powder was incorporated in distilled water in a proportion of 1:6 (*w*/*v*) to be extracted for 5 h at 95 °C (Section 1). Furthermore, the extraction of remains was performed employing distilled water in 1:6 ratios for 10 h at 95 °C (Section 2). Two sections for extracted solutions were cooled and mixed at room temperature. The mixed aqueous solution for extraction was filtrated by employing the Whatman No. 1 filter paper to obtain *S. aromaticum* extract. The condensation of the ultimate SAE was carried out using a void rotating evaporator (RV3VS002, IKA, Staufen, Germany) at 85 °C. Then, the extracts were reserved at a freezing condition at −55 °C until research.

The fresh beef loin and backfat from different steers were obtained from a local packing plant at 5 days post-slaughter. Beef loin from different steer was pooled and treated as a replication. Each loin was trimmed of any visible fat and connective tissues. The beef loin and backfat were minced twice separately using a meat grinder (YW456, LEM products, West Chester, OH, USA) containing a square pan fixed in the hole for 6-mm dimension. The total ground beef was prepared through similar formulates. The beef loin, backfat, and other ingredients were admixed completely at the right proportion utilizing a mixer (5K5SS, KitchenAid, Benton Harbor, MI, USA). The samples were formulated with 90.8% of beef loin, 8.0% of backfat, and 1.2% of sodium chloride (salt). The pre-weighed amount of BHT and SAE as formulations were then incorporated into the batter of ground meat and mixed entirely. Each type of ground beef was mixed triplicate for 2 min. Three types of ground beef were formulated regarding the preparation subsequently: (1) control, formulated no antioxidant; (2) T1, formulated by 0.02% of BHT; (3) T2, formulated by 0.1% of SAE. The formation of ground beef (40 g/ground beef) was carried out by hand. The ground beef was cooked by boiling at 90 °C for 30 min utilizing the water bath (BW-20G, Lab. Companion, Daejeon, Korea) and cooled at room temperature. All the cooked ground beef were packaged for each storage period employing a polyethylene packet (Thai Griptech Co. Ltd., Bangkok, Thailand). At last, the storage of cooked ground beef was performed in the freezing (−20 °C) condition for 0, 1, 2, 3, 4, 5, and 6 months to carry out the total experiment.

### 2.2. pH Value and Thawing Loss

The pH evaluation was carried out using the MP230 portative pH-meter (Mettler-Toledo, Greifensee, Switzerland) built in a glass electrode. By the addition of 30 mL distilled water to 3 g for samples, the homogenization was conducted for 25 s using the T-25 ULTRA-TURRAX homogenizer (Ultra-Turrax, IKA^®^, Staufen, Germany). The calibration of the pH-meter was performed employing three standard buffers such as pH 4.0, 7.0, and 9.0 at room temperature.

The thawing loss of samples was performed using Equation 1, following the method of Molette et al. [19] with minor modifications. The samples were stored at frozen (−20 °C) storage for specific periods. Then, the cooked ground beef was thawed throughout the night at refrigerated (4 °C) storage. The weight of samples was determined before and after thawing:

Thawing loss (%) = (T_1_ − T_2_)/T_1_ × 100 (1)

where, T_1_ is a weighing for the unthawed sample, and T_2_ is a weighing for thawed sample.

### 2.3. Lipid Oxidation

Thiobarbituric acid-reactive substance (TBARS) values for different samples were assessed following the procedure described by Cherian et al. [20] with minor modifications. The homogenization for the sample (3 g) was performed by the addition of 27 mL of perchloric acid (3.86%). After keeping at 4 °C for 60 min, the centrifugation of the homogenates was conducted for 10 min at 2000× *g* using a 1736R centrifuge (Labogene Co., Seoul, Korea). The supernatant was filtrated employing filtering papers of Whatman No. 4. Two mL TBA (20 mM) was inserted in 2 mL filtrate and kept in the dark at room temperature for 15 h. Then, the absorbances for such samples were measured at 531 nm spectrophotometrically (Cary 60 UV-Vis, Agilent Technologies Inc., Seoul, Korea). The TBARS were indicated as mg malondialdehyde per kg samples.

### 2.4. Volatile Compound Analysis

The volatile compound for cooked ground beef was analyzed employing gas chromatography along with mass spectrometry (GC-2010 Plus and GCMS-TQ 8030, Shimadzu, Tokyo, Japan) and using the method explained by Carballo et al. [11] with modifications. An aliquot of 1 g of ground beef was transferred into a 20 mL screw cap vial, and the vial was shaken at 30 °C for 25 min in the chromatograph autosampler to extract the volatile compound into the headspace. The injection volume of 1 mL was applied at 250 °C as injector temperature. The separation of the volatile compound was performed using the DB-WAX column (30 m × 0.25 mm id, 0.25 um film thickness; J and W Scientific, Santa Clara, CA, USA). The mass spectrometer was utilized to determine the affluence of the GC column. The ionization was carried out by applying 70 eV. The ion source temperature was 230 °C, and the ion interface temperature was 250 °C. The volatile compounds were determined by utilizing computed retention indices (RIs), GC/MS databases such as NIST 11 and Wiley 9 mass spectral library, and actual standard. The result for volatile compounds was stated in area units (AU) × 10^6^/g of the sample.

### 2.5. Instrumental Color Evaluation

The color value for different samples was measured by evaluating International Commission on Illumination lightness (L*), redness (a*), and yellowness (b*) employing a CR-400 Chroma Meter (Konica Minolta, Tokyo, Japan). To calibrate this colorimeter standard white plate was utilized (Y = 81.2; x = 0.3191; y = 0.3263). The assessment for every single sample was performed thrice. The polar coordinated chroma (C*) and hue angle (h°) values were evaluated through the equations, sequentially. The instrumental color evaluation was carried out by the suggestion of Minolta (1994) [21].

C* = (a* + b*)^1/2^ (2)

h° = tan^−1^(b*/a*) (3)

### 2.6. Statistical Analysis

Data were presented as mean values of triplicates with the standard error of means (SEMs). All evaluations were conducted in three replications at various times in the same location and planned randomly. The data were analyzed employing the software of SAS^®^ (version 9.3, SAS Institute, Inc., Cary, NC, USA, 2014) program. An analysis of variance (ANOVA) was carried out to calculate and compare the mean values for various parameters. A one-way ANOVA, which regards storage period and treatment as fixed effects, was used for analyzing the data acquired during storage. Duncan’s multiple range test (*p* < 0.05) was employed to evaluate the significant difference of means among the treatments.

## 3. Results and Discussion

### 3.1. pH Values and Thawing Loss

Table 1 showed the changes in pH values and thawing loss of cooked ground beef processed with BHT and SAE during storage. All samples showed an insignificant (*p* > 0.05) difference in pH values and thawing loss during all frozen storage periods. SAE- and BHT-treated samples revealed a non-significant difference for pH value compared with the control (*p* > 0.05). Mokhtar and Youssef [22] found that non-significant differences in pH value in BHT/BHA and SAE-added beef burger and the control during storage times (*p* > 0.05). Nonetheless, a significant (*p* < 0.05) decrease for pH values was reported in clove-formulated pork sausages compared with the control over the storage periods [23]. Such decline of pH could be owing to the development for more acid prompted by the generation of carbonic acid acted by increasing for carbon dioxide which reacted with moisture in meat products while keeping for storage [24]. The pH values increased at the end of storage in all treated samples. The pH value for meat products increased during frozen storage, which is due to the production of ammonia arising from amino acids deterioration as protein [3].

Nevertheless, thawing loss for all cooked ground beef increased significantly (*p* < 0.05) from the 1st to 6th month of storage. The results indicated that the storage period showed a significant effect (*p* < 0.05) on thawing loss of cooked ground beef while addition of antioxidants had no effect on this parameter. The results are in agreement with those of Molette et al. [19], who presented that the thawing loss for turkey breast muscle increased throughout storage time. Oliveira et al. [25] reported that thawing loss in meat products is potentially due to higher denaturation of proteins, which induces the reduction in functional characteristics of thawed meat as the water-holding capacity and gelling power of protein.

### 3.2. Lipid Oxidation

TBARS is the most important indication for lipid oxidation found in meat products. Modifications for malondialdehyde (MDA) concentration such as TBARS for cooked ground beef comprising several antioxidants throughout frozen storages are shown in Table 1. Antioxidants incorporation and storage periods exhibited significant (*p* < 0.05) effects on TBARS value. Maximum TBARS was observed for the control sample during entire storage periods. On the other hand, antioxidants treated samples presented a pronounced decrease in TBARS values. A significant increase in TBARS value was found in all cooked ground beef from the initial to end of the frozen storage period (*p* < 0.05). The primary value in the control sample was 0.62 mg MDA/kg, which expanded to 0.94 mg MDA/kg at the final storage time. However, the primary to the final TBARS values for BHT and SAE were varied from 0.10 to 0.18 mg MDA/kg and 0.05~0.13 mg MDA/kg, sequentially. The variation for TBARS increasing for BHT and SAE-added beef samples was numerically lower than the control from the primary to the final of the storage month. The control showed a significantly increased TBARS value in all storage periods compared with SAE and BHT-added beef samples (*p* < 0.05). This increased TBARS value of the control sample might be formed because of the production for higher MDA that is considered as a secondary product in the lipid oxidative process [26]. A non-significant (*p* > 0.05) difference of TBARS value was found among SAE- and BHT-formulated samples until 4 months of storage. Nevertheless, after 5 and 6 months of storage, SAE-incorporated samples presented significantly decreased TBARS values compared with BHT-treated samples (*p* < 0.05). This result indicated that SAE had greater efficiency for showing the lipid stability against oxidative process than BHT. The antioxidative efficiency for SAE can be due to the phenolic compound and the hydrogen atom donating capability for neutralizing free-radicals and preventing further oxidization [27]. The significant reduction in TBARS value was seen in pork patty [28], beef burger [22], chicken meats [26], and pork sausages [17] with added SAE. Furthermore, Mokhtar and Youssef [22] reported that SAE-treated beef burgers revealed a significantly lower TBARS value compared with BHT/BHA-treated beef burger during the storage period and such results indicated that SAE had increased effectiveness to restrain lipid oxidation in the beef burger in comparison with the BHT/BHA.

### 3.3. Volatile Compounds Analysis

**Figure 1:**
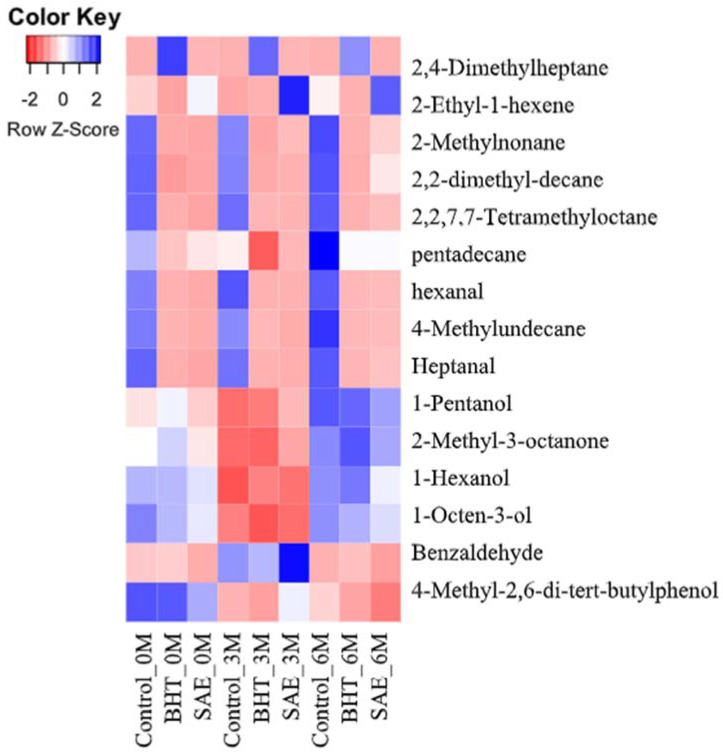
Heat map showing the changing patterns in volatile concentrations based on normalized intensity values across types of cooked ground beef and exhibition time. The volatile compounds were normalized against the abundance of internal standard 2-Methyl-1-pentanol. The red color indicates lesser abundance, and the green color indicates a higher abundance. Con: control; BHT: added 0.02% BHT; SAE: added 0.1% *Syzygium aromaticum* extract; 0M: 0 month of storage; 3M: 3 months of storage; 6M: 6 months of storage.

The key function of lipid oxidation in meat product to generate the secondary volatile components such as aldehydes, ketones, and alcohols, which may affect the fresh flavor or odor of meat and meat products and develop rancid odor [24]. The sum of 16 volatile compounds such as three aldehydes, seven hydrocarbons, one ketone, three alcohols, one phenol, and one benzene in cooked ground beef was detected and measured, and are shown in Figure 1 and Table 2. For visualizing the change in volatiles throughout the exhibition, the heat map (Figure 1) was designed for volatiles, which showed significant differences (*p* < 0.05). The heat map helped to esteem the changes for the intensity of volatiles through the exhibition period and amongst the antioxidants added samples. During the present analysis, three cooked ground beef such as the control, BHT-, and SAE-included samples indicated significant differences in the intensity level of volatiles. The colors of the heat map indicated that the control sample revealed more abundant for half of the volatiles (hexanal, heptanal, 2,2,7,7-tetramethyl-octane, 4-methyl-undecane, 1-pentanol, 1-hexanol, and 1-octen-3-ol) than BHT- and SAE-formulated samples during the end of the storage for 6 months (*p* < 0.05); however, the lowest abundant for benzaldehyde was found in SAE contained samples amongst all types of cooked ground beef (*p* < 0.05).

The majority of these volatiles such as aldehyde, hydrocarbon, ketone, and alcohol are probably generated through lipid degradation [12], which can occur because of the thermic oxidation at the time of beef cooking and volatile extracting. The addition of antioxidants to cooked ground beef revealed significant effects on the majority of the particular volatiles (mostly consisting of lipid oxidized secondary product) among the above-mentioned families. The significant (*p* < 0.05) reduction for hexanal, heptanal, octanal, 2, 4-dimethyl-heptane, 2-methyl-3-octanone, 1-pentanol, 1-hexanol, and 1-octen-3-ol was found in SAE- and BHT-formulated samples compared with the control after month 6 of storage (Table 2). Especially, the amounts of aldehydes in cooked ground beef increased during storage, but SAE- and BHT-added ground beef produced fewer aldehydes than control. The increase in aldehydes in cooked meat should be caused by lipid oxidation under aerobic conditions during storage, but the treated samples did not affect the amounts of aldehydes during storage. Hexanal was the major volatile aldehyde and the production of aldehydes agreed well with TBARS data (Table 1). This result is in accordance with those of Yang et al. [11], who recorded that hexanal is an important volatile aldehyde and the increased hexanal level is related to the increased value of TBARS in beef, indicating that the control ground beef showed a higher TBARS value with an increased level of hexanal. Soriano et al. [24] reported that the control pork patties showed a higher level of hexanal as aldehyde in comparison with the natural antioxidant contained pork patties. Calkins and Hodgen [29] also reported that hexanal is generated from the oxidation of fatty acid and is a well-known off-flavor observed in meat products. As for 1-pentanol and benzaldehyde, the SAE-added beef sample presented substantially (*p* < 0.05) reduced levels compared with all other beef samples on month 6. Therefore, the results indicate that the antioxidants retard the thermic decomposition of lipid and decreased the lipid oxidized volatile compounds level. These effects might be described with the capability of natural antioxidants for scavenging lipid radical, leading to the prevention of free radicals chain reactions for lipid oxidization [30]. The reducing effects of added natural antioxidants on the level of lipid-originated volatile constituents for cooked lamb patties were already reported by Carballo et al. [12], who observed a decreased level for aldehydes, hydrocarbons, ketones, and alcohols in patties. Their study is similar to the current analysis and indicated increased lipid stability against oxidation because of the presence of natural antioxidants in cooked ground beef.

The non-significant (*p* > 0.05) differences among all cooked ground beef were observed for 2, 2, 7, 7-tetramethyl-octane, pentadecane, 2-methyl-nonane, and 4-methyl-undecane for entire storage periods. However, from month 0 to 6 of storage, the content of 2, 4-dimethyl-heptane in control and BHT-formulated ground beef, 2-ethyl-1-hexene in BHT-treated samples, and 2-ethyl-1-hexene in SAE-treated ground beef were significantly (*p* < 0.05) increased.

Alcohol as a volatile compound is generated by the deterioration of triglyceride and phospholipid, the oxidation of aldehyde, and bacterial activities [3]. This alcohol compound may also be the basic material for oxidation. The alcohols such as 1-pentanol, 1-hexanol, and 1-octen-3-ol were significantly higher for the control compared with antioxidants treated cooked beef samples (*p* < 0.05). Therefore, the changes in reduced level of volatiles components by the addition of SAE indicate the poor lipid oxidation of cooked ground beef at frozen storage period because of the antioxidant properties of SAE. Finally, SAE not only decreased the generation of volatiles component owing to the oxidation process but also showed an impressive effect on the quality trait in cooked ground beef because it showed a lowered level of lipid oxidation in cooked ground beef. Nonetheless, the similar findings were seen by other authors in cooked meat and meat products employing several plant extracts because of their inhibiting effects on lipid oxidization [31].

### 3.4. Color Values

The color is the most appreciated parameter for meat products and may influence the consumers to immediately purchase or refuse the meat product after observance [24]. Table 3 presents the effect of added antioxidants on the color values for cooked ground beef assessed each month for frozen storage. After 6 months of storage, color values such as redness (a*), yellowness (b*), chroma (C*), and hue angle (h^°^) did not show significant changes amongst all cooked ground beef (*p* > 0.05). Nevertheless, SAE-added ground beef presented significantly reduced lightness (L*) value compared with BHT-treated ground beef after 6 months of frozen storage (*p* < 0.05), and the control and antioxidants as SAE-added samples were not significantly different for L* value (*p* > 0.05). This decrease in L* value for SAE-treated samples might have resulted because of the existence of purple pigments in clove extracts [32]. The L* value for SAE-formulated samples was significantly decreased from 0 to 6 months for storage (*p* < 0.05); nonetheless, no significant change for L* value was seen in BHT-formulated samples and control (*p* > 0.05). From month 0 to month 6, a* and h^°^ values for all cooked ground beef were substantially (*p* < 0.05) increased and reduced, respectively. It is observed that the antioxidants such as SAE and BHT can show protective impacts against color deterioration in cooked ground beef with the progress of the storage period. Zhang et al. [17] stated that the preventive effect for SAE against color deterioration in meat products was observed because of the antioxidant activities of phenolic components. Yousuf and Srivastava [33] mentioned that reduced hue angle (h^°^) value was connected with reduced deterioration for red color, specifying that all antioxidants-added patties presented decreased degradation of color with the increase of storage time. The b* values for BHT-included samples lowered substantially (*p* < 0.05) from 0 to 6 months of frozen storage; nonetheless, no significant (*p* > 0.05) changes in b* values were found in the control and SAE-treated samples. However, a non-significant change for C* value was observed for all kinds of cooked ground beef during storage (*p* > 0.05). The findings are in good agreement with Jin et al. [23], who presented that the inclusion of 0.1% SAE to pork sausage led to the non-significant differences for a*and b* values during the final storage compared with control sausage. Zhang et al. [17] presented that SAE-added sausage showed substantially reduced L* and increased a* values and an insignificant difference in b* values compared with control sausage.

## 4. Conclusions

The noticeable outcome found in cooked ground beef after addition of BHT and SAE reduced lipid oxidation in comparison with the control during frozen storage of 6 months. Incorporation for SAE and BHT strongly inhibit the formation of volatile compounds due to oxidation. Here, SAE proved itself as an active natural antioxidant compared with BHT by improving product oxidative stability during storage. Among all treatments, antioxidants had an insignificant effect on each color values except yellowness. Therefore, the results pointed out that the use of SAE as a natural antioxidant in place of BHT in cooked ground beef can minimize lipid oxidation and volatiles formation during long term frozen storage in industrial and small-scale retail vendors.

## Figures and Tables

**Table 1:** The pH, thawing loss, and TBARS values of cooked ground beef with different antioxidant additives at frozen storage.

	Storage Month	Con	BHT	SAE	SEM
pH	0	5.80 ^Ba^	5.82 ^Ba^	5.80 ^Ba^	0.03
	1	5.85 ^Ba^	5.87 ^ABa^	5.87 ^Ba^	0.02
	2	5.88 ^ABa^	5.89 ^ABa^	5.90 ^ABa^	0.02
	3	5.87 ^ABa^	5.87 ^Ba^	5.85 ^Ba^	0.01
	4	5.87 ^ABa^	5.86 ^Ba^	5.85 ^Ba^	0.03
	5	5.89 ^ABa^	5.89 ^ABa^	5.90 ^ABa^	0.02
	6	6.01 ^Aa^	6.01 ^Aa^	6.03 ^Aa^	0.10
	SEM	0.03	0.03	0.04	
Thawing loss (%)	1	0.05 ^Ca^	0.05 ^Ba^	0.05 ^Ba^	0.00
	2	0.06 ^Ca^	0.09 ^Ba^	0.07 ^Ba^	0.01
	3	0.10 ^BCa^	0.09 ^Ba^	0.09 ^Ba^	0.02
	4	0.17 ^BCa^	0.20 ^ABa^	0.36 ^ABa^	0.10
	5	0.35 ^ABa^	0.43 ^Aa^	0.83 ^Aa^	0.16
	6	0.55 ^Aa^	0.45 ^Aa^	0.79 ^Aa^	0.13
	SEM	0.05	0.05	0.11	
TBARS (mg MDA/kg sample)	0	0.62 ^Da^	0.10 ^BCb^	0.05 ^Cb^	0.04
	1	0.67 ^CDa^	0.10 ^Cb^	0.06 ^Cb^	0.02
	2	0.77 ^BCDa^	0.12 ^ABCb^	0.08 ^BCb^	0.03
	3	0.80 ^ABCa^	0.12 ^ABCb^	0.08 ^BCb^	0.02
	4	0.85 ^ABa^	0.15 ^ABCb^	0.11 ^ABb^	0.02
	5	0.90 ^ABa^	0.17 ^ABb^	0.11 ^ABc^	0.01
	6	0.94 ^Aa^	0.18 ^Ab^	0.13 ^Ac^	0.01
	SEM	0.05	0.02	0.01	

^a–c^ Mean values in the same row with different letters presented significant differences (*p* < 0.05). ^A–D^ Mean values in the same column with different letters presented significant differences (*p* < 0.05). Con: control; BHT: added 0.02% BHT; SAE: added 0.1% *Syzygium aromaticum* extract. SEM: standard error of mean.

**Table 2:** Volatile compounds of cooked ground beef with different antioxidant additives at frozen storage.

	Storage Month	Con	BHT	SAE	SEM
Aldehydes					
Hexanal	0	19.68 ^Aa^	2.62 ^Ab^	2.12 ^Ab^	0.92
	3	17.62 ^Aa^	2.47 ^Ab^	1.08 ^Ac^	0.70
	6	21.95 ^Aa^	3.07 ^Ab^	1.31 ^Ab^	1.40
	SEM	2.08	0.75	0.52	
Heptanal	0	0.67 ^Aa^	0.03 ^Ab^	0.00 ^Bb^	0.04
	3	0.60 ^Aa^	0.07 ^Ab^	0.00 ^Bb^	0.05
	6	0.71 ^Aa^	0.08 ^Ab^	0.04 ^Ab^	0.05
	SEM	0.12	0.03	0.00	
Octanal	0	0.57 ^Aa^	0.35 ^Aa^	0.03 ^Aa^	0.22
	3	0.37 ^Aa^	0.05 ^Ab^	0.04 ^Ab^	0.04
	6	0.39 ^Aa^	0.07 ^Ab^	0.05 ^Ab^	0.04
	SEM	0.14	0.12	0.01	
Hydrocarbons					
2,2,7,7-Tetramethyl-octane	0	3.97 ^Aa^	3.91 ^Aa^	7.90 ^Aa^	1.27
	3	1.99 ^Aa^	1.82 ^Ba^	2.90 ^Aa^	0.36
	6	2.27 ^Aa^	1.86 ^Ba^	1.91 ^Aa^	0.27
	SEM	0.49	0.39	1.31	
Pentadecane	0	2.96 ^ABa^	3.34 ^ABa^	4.86 ^Aa^	0.66
	3	1.90 ^Ba^	2.06 ^Ba^	2.25 ^Ba^	0.17
	6	4.74 ^Aa^	4.60 ^Ba^	5.17 ^Aa^	0.76
	SEM	0.61	0.51	0.56	
2-Methyl-nonane	0	0.75 ^Ba^	0.77 ^ABa^	1.95 ^Aa^	0.62
	3	1.86 ^Aa^	1.64 ^Aa^	2.77 ^Aa^	0.36
	6	0.59 ^Ba^	0.68 ^Ba^	0.66 ^Aa^	0.07
	SEM	0.22	0.23	0.64	
2,4-Dimethyl- heptane	0	0.62 ^Ba^	0.37 ^ABb^	0.47 ^Ab^	0.07
	3	0.45 ^Ba^	0.17 ^Bb^	0.16 ^Bb^	0.05
	6	0.97 ^Aa^	0.49 ^Ab^	0.43 ^Ab^	0.08
	SEM	0.09	0.06	0.06	
4-Methyl-undecane	0	0.49 ^Aa^	0.58 ^Aa^	0.91 ^Aa^	0.13
	3	0.21 ^Aa^	0.20 ^Ba^	0.36 ^Ba^	0.05
	6	0.72 ^Aa^	0.82 ^Aa^	0.63 ^ABa^	0.14
	SEM	0.14	0.09	0.10	
2-ethyl-1-hexene	0	0.88 ^Aa^	0.87 ^ABa^	1.16 ^Aa^	0.15
	3	0.43 ^Ba^	0.51 ^Ba^	0.56 ^Ba^	0.08
	6	0.95 ^Ab^	0.99 ^Ab^	1.29 ^Aa^	0.06
	SEM	0.09	0.11	0.12	
Ketone					
2-methyl-3-octanone	0	0.84 ^Aa^	0.04 ^Ab^	0.00 ^Ab^	0.06
	3	0.77 ^Aa^	0.05 ^Ab^	0.00 ^Ab^	0.03
	6	1.11 ^Aa^	0.04 ^Ab^	0.01 ^Ab^	0.06
	SEM	0.17	0.02	0.00	
Alcohols					
1-Pentanol	0	0.69 ^Aa^	0.08 ^Ab^	0.02 ^Ab^	0.03
	3	0.82 ^Aa^	0.09 ^Ab^	0.03 ^Ab^	0.08
	6	0.81 ^Aa^	0.10 ^Ab^	0.04 ^Ac^	0.02
	SEM	0.11	0.03	0.01	
1-Hexanol	0	0.11 ^Aa^	0.02 ^Ab^	0.02 ^Ab^	0.00
	3	0.11 ^Aa^	0.02 ^Ab^	0.01 ^Ab^	0.01
	6	0.12 ^Aa^	0.02 ^Ab^	0.01 ^Ab^	0.00
	SEM	0.01	0.00	0.00	
1-Octen-3-ol	0	0.64 ^Aa^	0.07 ^Ab^	0.04 ^Ab^	0.02
	3	0.61 ^Aa^	0.08 ^Ab^	0.04 ^Ab^	0.04
	6	0.67 ^Aa^	0.09 ^Ab^	0.04 ^Ab^	0.03
	SEM	0.08	0.03	0.01	
Phenol					
4-Methyl-2,6-di-tert-butylphenol	0	0.22 ^Ab^	32.25 ^Aa^	0.22 ^ABb^	1.72
	3	0.21 ^Ab^	27.41 ^Aa^	0.39 ^Ab^	0.52
	6	0.16 ^Ab^	22.73 ^Aa^	0.08 ^Bb^	1.16
	SEM	0.08	4.27	0.07	
Benzene					
Benzaldehyde	0	0.31 ^Aa^	0.25 ^Ab^	0.09 ^Ac^	0.03
	3	0.26 ^Aa^	0.27 ^Aa^	0.10 ^Ab^	0.04
	6	0.34 ^Aa^	0.26 ^Ab^	0.11 ^Ac^	0.04
	SEM	0.03	0.04	0.02	

Values showed as AU ×10^6^/g of patties sample. ^a–c^ Mean values in the same row with different letters presented significant differences (*p* < 0.05). ^A,B^ Mean values in the same column with different letters presented significant differences (*p* < 0.05). Con: control; BHT: added 0.02% BHT; SAE: added 0.1% *Syzygium aromaticum* extract. SEM: standard error of mean. AU: Arbitrary Area Unit resulted for chromatographic ion count.

**Table 3:** Color of cooked ground beef with different antioxidant additives at frozen storage.

	Storage Month	Con	BHT	SAE	SEM
Lightness (L*)	0	48.09 ^Aa^	49.43 ^Aa^	47.94 ^Aa^	0.87
	1	46.69 ^Aab^	47.11 ^ABa^	45.20 ^Bb^	0.49
	2	46.61 ^Aa^	46.66 ^Ba^	45.64 ^Ba^	1.06
	3	46.32 ^Aa^	46.61 ^Ba^	45.43 ^Ba^	0.8
	4	47.40 ^Aab^	48.47 ^ABa^	45.10 ^Bb^	0.68
	5	47.96 ^Aa^	47.26 ^ABab^	44.96 ^Bb^	0.71
	6	45.55 ^Aab^	47.86 ^ABa^	45.03 ^Bb^	0.68
	SEM	0.76	0.63	0.63	
Redness (a*)	0	4.42 ^Bb^	4.11 ^Bb^	5.26 ^Ba^	0.34
	1	5.21 ^Ba^	5.01 ^Ba^	5.07 ^Ba^	0.21
	2	7.94 ^Aa^	8.50 ^Aa^	8.35 ^Aa^	0.43
	3	7.55 ^Aa^	8.12 ^Aa^	8.09 ^Aa^	0.69
	4	7.49 ^Aa^	7.65 ^Aa^	8.08 ^Aa^	0.28
	5	7.25 ^Aa^	7.98 ^Aa^	7.70 ^Aa^	0.3
	6	8.04 ^Aa^	8.12 ^Aa^	8.38 ^Aa^	0.5
	SEM	0.43	0.32	0.32	
Yellowness (b*)	0	14.84 ^Aa^	15.11 ^Aa^	14.93 ^Aa^	0.52
	1	14.40 ^ABb^	15.46 ^Aa^	15.70 ^Aa^	0.28
	2	13.31 ^Ba^	13.61 ^Ba^	14.61 ^Aa^	0.38
	3	13.71 ^ABa^	13.76 ^Ba^	14.31 ^Aa^	0.46
	4	13.37 ^Bb^	13.68 ^Bb^	14.66 ^Aa^	0.24
	5	13.58 ^Bb^	13.73 ^Bb^	14.80 ^Aa^	0.3
	6	13.78 ^ABa^	13.81 ^Ba^	14.89 ^Aa^	0.43
	SEM	0.34	0.4	0.39	
Chroma (C*)	0	15.52 ^Aa^	15.68 ^Aa^	15.85 ^Aa^	0.45
	1	15.34 ^Ab^	16.26 ^Aab^	16.53 ^Aa^	0.27
	2	15.52 ^Ab^	16.09 ^Aab^	16.87 ^Aa^	0.31
	3	15.59 ^Ab^	16.03 ^Aab^	16.32 ^Aa^	0.26
	4	15.34 ^Ab^	15.69 ^Ab^	16.75 ^Aa^	0.27
	5	15.42 ^Ab^	15.89 ^Aab^	16.73 ^Aa^	0.28
	6	16.00 ^Aa^	16.04 ^Aa^	17.14 ^Aa^	0.54
	SEM	0.31	0.36	0.36	
Hue angle (*h°*)	0	73.38 ^Aab^	74.76 ^Aa^	70.61 ^Ab^	1.54
	1	70.17 ^Ab^	72.13 ^Aa^	72.11 ^Aab^	0.7
	2	59.17 ^Ba^	57.80 ^Ba^	60.27 ^Ba^	1.7
	3	60.90 ^Ba^	59.41 ^Ba^	60.24 ^Ba^	1.68
	4	60.70 ^Ba^	60.75 ^Ba^	61.16 ^Ba^	0.92
	5	61.86 ^Ba^	59.83 ^Ba^	62.53 ^Ba^	1
	6	59.83 ^Ba^	59.58 ^Ba^	60.53 ^Ba^	1.43
	SEM	1.6	1.15	1.21	

^a–c^ Mean values in the same row with different letters presented significant differences (*p* < 0.05). ^A–C^ Mean values in the same column with different letters presented significant differences (*p* < 0.05). Con: control; BHT: added 0.02% BHT; SAE: added 0.1% *Syzygium aromaticum* extract; SEM: standard error of mean.

## Data Availability

Data is contained within the article.
